# *De novo* transcriptome assembly of the green alga *Ankistrodesmus falcatus*

**DOI:** 10.1371/journal.pone.0251668

**Published:** 2021-05-14

**Authors:** Rachel A. Schomaker, Jeffry L. Dudycha

**Affiliations:** Department of Biological Sciences, University of South Carolina, Columbia, SC, United States of America; International Centre for Genetic Engineering and Biotechnology, INDIA

## Abstract

*Ankistrodesmus falcatus* is a globally distributed freshwater chlorophyte that is a candidate for biofuel production, is used to study the effects of toxins on aquatic communities, and is used as food in zooplankton research. Each of these research fields is transitioning to genomic tools. We created a reference transcriptome for of *A*. *falcatus* using NextGen sequencing and *de novo* assembly methods including Trinity, Velvet-Oases, and EvidentialGene. The assembled transcriptome has a total of 17,997 contigs, an N50 value of 2,462, and a GC content of 64.8%. BUSCO analysis recovered 83.3% of total chlorophyte BUSCOs and 82.5% of the eukaryotic BUSCOs. A portion (7.9%) of these supposedly single-copy genes were found to have transcriptionally active, distinct duplicates. We annotated the assembly using the dammit annotation pipeline, resulting in putative functional annotation for 68.89% of the assembly. Using available rbcL sequences from 16 strains (10 species) of *Ankistrodesmus*, we constructed a neighbor-joining phylogeny to illustrate genetic distances of our *A*. *falcatus* strain to other members of the genus. This assembly will be valuable for researchers seeking to identify *Ankistrodesmus* sequences in metatranscriptomic and metagenomic field studies and in experiments where separating expression responses of zooplankton and their algal food sources through bioinformatics is important.

## Introduction

*Ankistrodesmus* is a genus of unicellular, freshwater algae in the family Selenastraceae. These chlorophytes are model organisms for studying cellular physiology in phytoplankton because they are able to survive under many different growth conditions and exhibit rapid growth rates compared to other algal species. For example, Brown and Weis, studied the metabolic interconnections between photosynthesis and respiration in *A*. *braunii* [1], and Shatilov, *et al*. used the same species to further our understanding of chloroplast-encoded enzymatic activity within the cell [2]. More recently, Asselborn, *et al*. showed the potential effects of insecticides on phytoplankton communities [3] and Skorupskaite, *et al*. determined the best ways to disrupt cell membranes for biofuel production [4], both using *Ankistrodesmus* as models.

*Ankistrodesmus falcatus* is globally distributed in both lentic freshwaters and large, slow-moving rivers [5]. Field studies have shown that *A*. *falcatus* can be seasonally dominant or otherwise important in freshwater ecosystems [6–8]. Like other *Ankistrodesmus* species, *A*. *falcatus* is a prime candidate species for biofuel production because of its high lipid, pigment, and polysaccharide content [9–13]. It is also a model organism for studying cell growth and division and is a proxy for determining how algal communities respond to heavy metal pollution in freshwater systems [14, 15].

*A*. *falcatus* is often an experimental food source for *Daphnia*, which are freshwater microcrustaceans found in lakes and ponds across the globe. *Daphnia* are ecologically important in the ecosystems they inhabit because they are prey sources for many higher-level fish and invertebrate consumers [16, 17] and they also act as consumers, preying upon algal primary producers in these systems [18, 19]. Additionally, they are often used as indicator species for determining the health and water quality of the aquatic ecosystems they inhabit [20, 21]. *Daphnia* are frequently used as experimental models because they are easy to maintain in culture, have a host of publicly available genetic information, and reproduce asexually, making them ideal for research topics where sexual reproduction may be a confounding factor. *Daphnia* fed *A*. *falcatus* have been used as models on research topics as diverse as consumer-resource interactions, [22–26] the evolution and genetics of aging, [27–29] disease ecology, [30–33] sensory biology, [34] local adaptation, [35–38] developmental ontogeny, [39] and nutritional physiology [40]. While microarray studies of consumer responses to dietary variation could be done in the absence of genomic data for the resource (e.g., [41]), the shift to NextGen sequencing for transcriptomics will require bioinformatic approaches to distinguish responses of the consumer from those of the consumed. The advent of feeding-based functional genetics for *Daphnia* [42, 43] will also benefit from better understanding of the genetics of algae used for basic diets.

One reference transcriptome for the genus *Ankistrodesmus* is currently available. Castro, *et al*. [44] used *Ankistrodesmus sp*. UCP0001 to characterize potential fatty acid biosynthesis pathways using transcriptomics, which is useful information for biofuel production. Thanh, *et al*. [45, 46] developed a set of expressed sequence tags and characterized ribulose-1,5-bisphosphate carboxylase/oxygenase (RuBisCo) using *A*. *convolutus*. However, this is the extent of genetic and genomic information available for this genus. Additionally, the *Ankistrodesmus* phylogeny is poorly known, and thus it is difficult to ascertain whether genetic differences from the currently available transcriptomic data would be too great for studies involving *A*. *falcatus* to be reliable. Studies which have investigated the phylogenetic relationships for other members of the Selanastraceae family and the *Ankistrodesmus* genus suggest that species within the *Ankistrodesmus* genus exhibit typical variation for the Selenastraceae [13, 47, 48]. Garcia da Silva *et al*. [48] created multiple phylogenies of the Selanastraceae family using the RuBisCo subunits and 18s rDNA, including various strains of *A*. *fusiformis*, *A*. *stipitatus*, *A*. *fasciculatus*, *A*. *spiralis*, and *A*. *arcutatus*, placing them in one or two clades depending on which gene was analyzed. Singh, *et al*. [13] created a phylogenetic tree that included multiple *A*. *falcatus* strains, two different *Ankistrodesmus sp*. strains, and *A*. *convolutus*, and inferred that *A*. *convolutus* is the most distantly related species. This is unsurprising, given that *A*. *convolutus* had been synonymized with *Monoraphidium convolutum* previously [49]. Overall, the lack of known genetic distance between these species and the high variation in phylogenetic groupings of the *Ankistrodesmus* genus in general suggests that it is important to increase the genomic information available for future studies.

We aimed to create a high-quality, publicly available *de novo* reference transcriptome of *A*. *falcatus*. This reference transcriptome will facilitate experimental transcriptomics of *A*. *falcatus* itself and its invertebrate consumers, metagenomic studies of natural freshwater communities, and comparative sequence analysis of chlorophytes and higher-order taxa.

## Materials and methods

### Strain source

**Fig 1:**
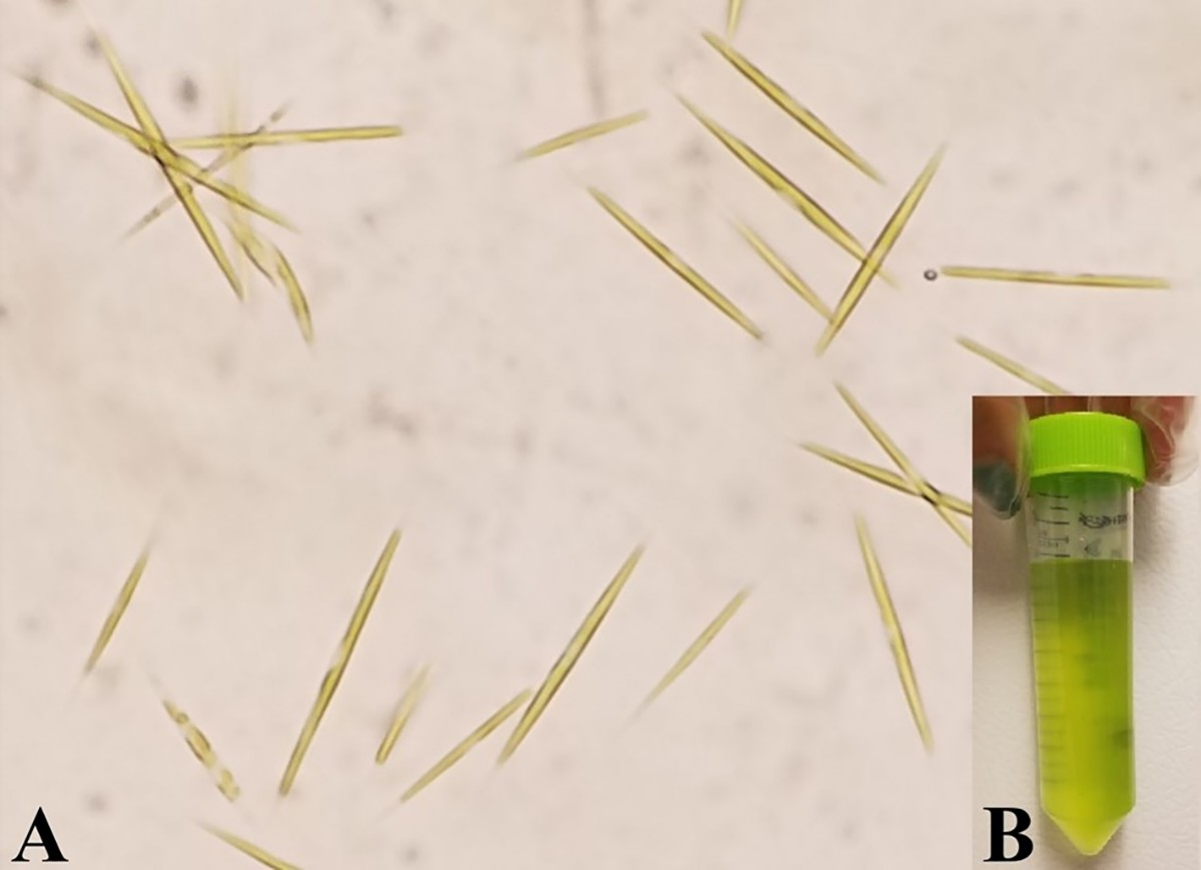
A–*A*. *falcatus* viewed under 40X magnification settled in a 1.0mL Sedgewick Rafter counting chamber (Wildco 1801-A10). B–Sample of *A*. *falcatus* taken from semi-continuous batch culture.

We obtained our strain of *A*. *falcatus* (Fig 1) from the lab of A. J. Tessier, who originally acquired it in the late 1970s from the lab of C. E. Goulden at the Academy of Natural Sciences in Philadelphia, PA, USA. The strain’s provenance prior to that is unknown. The earliest known published work with the strain is Goulden and Hornig, [22] in which the authors state that the strain has an unknown origin. Here, we designate this strain AJT.

### Growth conditions

We grew *A*. *falcatus* in semi-continuous culture under a 24 h:0 h L:D photoperiod with a light intensity of ~100 μmol photons/m^-2^s^-1^ PAR, from fluorescent lamps (CH Lighting F32T8/841/ECO) arranged laterally on one side of the culture vessels. Cultures are left to grow at ambient room temperatures (20–23°C). We grew cultures in ASM-1 freshwater algal medium [50] with added vitamins. The added vitamin solution included biotin, thiamine, pyridoxine, calcium pantothenate, B12, nicotinic acid, nicotinamide, folic acid, riboflavin, and inositol at the concentrations specified in Goulden and Hornig [22]. The culture was kept in 5L bottles set up with constant aeration and stirring at 400 rpm to prevent settling. Samples for RNA extractions were taken when the cultures were in exponential phase.

### RNA extraction protocol

We extracted total RNA from 100 mL of the *A*. *falcatus* stock using a modified procedure for the Qiagen RNeasy Plant Mini Kit RNA extraction protocol. We split the 100 mL sample into two 50 mL aliquots in 50 mL centrifuge tubes and then spun them down at 7000 rpm (5927g; Beckman Coulter J2-21 centrifuge; JA-20 rotor) for 15 minutes. After supernatant was removed, we transferred the pellets to two 2-mL centrifuge tubes. These tubes were spun at 5000 rpm (2340g; Eppendorf AG centrifuge 5424; Eppendorf rotor FA-45-24-11 5424/5424R) for 10 minutes. We again removed the supernatant, and then froze the pellets in liquid nitrogen. Once frozen, we added 450 μL of Buffer RLT with added β-mercaptoethanol (prepared by adding 10 μL β-mercaptoethanol to 1 mL of Buffer RLT) to each tube, and disrupted the cells using a handheld tissue homogenizer. We used the standard Qiagen RNeasy Plant Mini Kit RNA extraction procedure for the remainder of the extraction process.

Once extracted, we checked the purity of the RNA using a Nanodrop 2000 and obtained the concentration with a Qubit 4 Fluorometer. We checked the integrity of the RNA by running a sample of the extracted RNA on a 2% agarose gel at 60V for 1 hr. RNA samples were considered good quality if the 260/280 and 260/230 ratios were greater than 1.8 and clear rRNA bands could be observed on the gel without signs of degradation. The sample with the best quality control metrics overall was sent to Vanderbilt Technologies for Advanced Genomics (VANTAGE) for 150bp paired-end (PE) NovaSeq 6000 sequencing targeting 100 million reads per sample. Library preparation was performed at VANTAGE using the Illumina Tru-seq RNA sample library prep kit.

### Transcriptome assembly

We checked the quality of the raw reads with FastQC [51]. Reads were trimmed and adapter sequences were removed using Trimmomatic [52] with the following parameters: ILLUMINACLIP:TreSeq3-PE.fa:2:30:10 HEADCROP:20. After Trimmomatic, 76.82% of the raw reads (38,277,563 out of 49,830,437 paired reads) remained, and we used these for the transcriptome assembly.

We first created a transcriptome with Trinity [53]. Then, we created several assemblies using kmer lengths of 35, 45, 55, 65, 75, 85, and 95 with Velvet-Oases [54]. The Velvet-Oases assemblies were merged to create one final Velvet-Oases assembly. We combined the Trinity final Velvet-Oases assemblies with EvidentialGene mRNA transcript assembly software (EviGene; [55]) with a kmer length of 75. We used EviGene to correct for the various biases attributed to different assemblers. Additionally, EviGene is useful for pulling out potential isoforms and splice variants of each gene, and separating these potential variations into an independent ‘alternative’ file so that the final assembly is less likely to be full of gene duplicates or isoforms, increasing the confidence that each contig that remains is indeed a unique gene. This ensured that we were left with the most comprehensive and accurate transcriptome assembly across both assembly methods.

### Quality control and statistics

We removed any remaining rRNA sequences from the final assembly by downloading the small and large rRNA subunits for *A*. *falcatus* from the SILVA database [56] and blasting these sequences against our *A*. *falcatus* assembly. Only 6 contigs came back with hits as rRNA subunit sequences and these were removed from the final assembly. We then used Benchmarking Universal Single Copy-Orthologs (BUSCO, version 3) to assess the completeness of the transcriptome by searching our assembly against the BUSCO Chlorophyta_odb10 (creation date: 2017-12-01) and the Eukaryota_odb9 (creation date: 2016-11-02) datasets [57]. We used TransRate [58] to obtain descriptive statistics and to assess the overall quality of the transcriptome assembly. We considered any contigs that had a “good” read mapping percent (“p_good” in the TransRate contig result file) of 0 to be poor quality and removed these contigs from the final assembly.

### Gene annotation

We used the *de novo* transcriptome annotator dammit [59] to annotate our final assembly. This pipeline uses Transdecoder to build gene models and then searches the Pfam-A, Rfam, OrthoDB, and uniref90 protein databases for annotation information with an E-value cutoff of 1x10^-5^. The putative transcripts were also run through InterProScan to obtain a broader sense of functional annotations.

### Genetic distance to other species

Our transcriptome produced a sequence for rbcL (ribulose bisphosphate carboxylase, large subunit) from *A*. *falcatus* strain AJT. Since no phylogeny for the genus is available, we sought to evaluate the genetic distance from other *Ankistrodesmus* species using rbcL. We downloaded all available *Ankistrodesmus* rbcL sequences from the NCBI nucleotide database, including one of *A*. *falcatus*. We aligned the sequences using MUSCLE as implemented in MEGA X (Kumar, et al. 2018) [60]. We visualized genetic distances by creating a neighbor-joining tree [61] and tested it with 500 bootstrap replicates, again using MEGA X.

## Results

### Transcriptome assembly statistics

After quality control, our assembly had 17,997 contigs with an average contig length of 1,737bp and a GC content of 64.8%. The N50 length was 2,462bp and the N70 was 1,726bp (Table 1). This is a substantial improvement over the only available reference transcriptome for *Ankistrodesmus* (an unknown species with a strain designator of UCP0001), which had an N50 of 1,038bp and an average contig length of 508bp [44]. Differences in sequencing depth, assembly methods, and species’ biological variation could all contribute to these differences in assembly metrics.

**Table 1:** Transcriptome summary statistics.

Total Raw Paired End Reads	49,830,437
Total Assembled Contigs	17,997
Total Assembled Bases	31,266,666
Mean Contig Length	1,736.71
Contig N50 value (nt)	2,462
Contig N70 value (nt)	1,726
GC Content (%)	64.8

BUSCO results recovered 82.5% (250 of the 303 groups) of the eukaryote database. When using the chlorophyte database, we recovered 83.3% (1805 of 2168 groups) BUSCO groups, with only 7.9% duplicated and 6.5% fragmented (Table 2).

**Table 2:** BUSCO results breakdown of the completed *A*. *falcatus* assembly against the eukaryote and chlorophyte databases. The eukaryote BUSCO results can be summarized as C:82.5%[S:78.9%,D:3.6%], F:6.6%, M:10.9%, n:303. The chlorophyte BUSCO results can be summarized as C:83.3%[S:75.4%,D:7.9%], F:6.5%, M:10.2%, n:2168.

BUSCO Category	Eukaryote Database	Chlorophyte Database
Complete BUSCOs (C)	239	1805
Complete and single-copy BUSCOs (S)	216	1634
Completed and duplicated BUSCOs (D)	23	171
Fragmented BUSCOs (F)	20	142
Missing BUSCOs (M)	33	221
Total BUSCO groups searched	303	2168

C = Complete; S = Complete and single-copy; D = Complete and duplicated; F = Fragmented; M = Missing.

We used TransRate to examine the alignment and read mapping characteristics of the final assembly. The TransRate results showed that a total of 79.5% of the total reads mapped back to our final assembly.

### Gene annotation

The dammit pipeline recovered 68.89% (12,399 out of 17,997) transcript annotations that were homologous to proteins across the Pfam-A, Rfam, OrthoDB, and uniref90 databases, which is comparable to the currently available *Ankistrodesmus* transcriptome. Only 9 of these recovered annotations came back as hypothetical proteins, and the remaining 31.11% of transcripts did not have an annotation hit across the protein databases.

**Fig 2:**
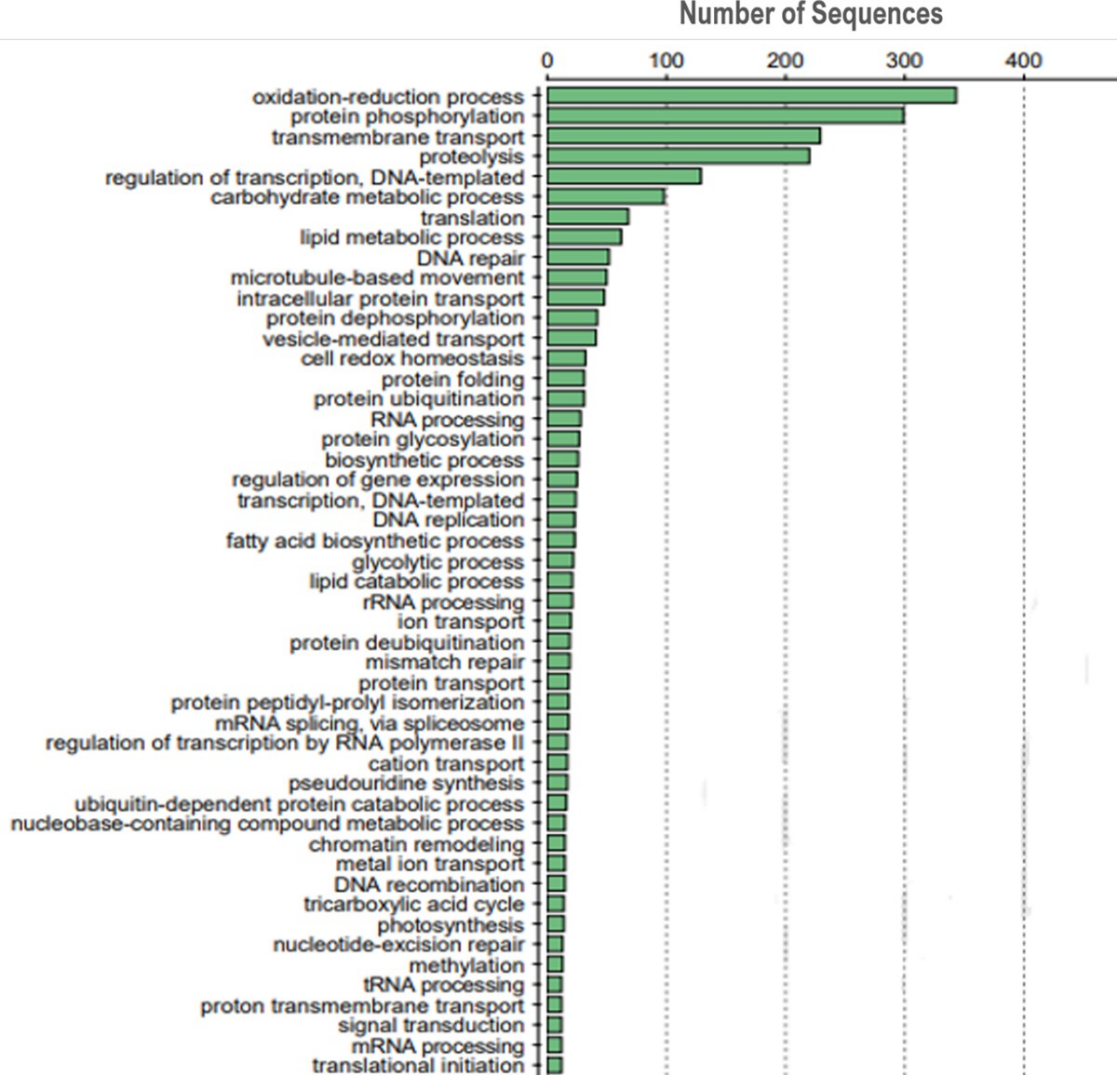
Gene ontology results for the biological processes annotated within the *A*. *falcatus* assembly.

We used InterProScan to obtain broad functional groupings along with the dammit annotation output to confirm that annotations that we expected to see in photosynthetic unicellular eukaryotes were present. These results suggested that the greatest proportion of the annotated genes were related to oxidation-reduction biological processes (including photosynthesis-related functions and electron transport), protein phosphorylation, transmembrane transport, lipid and carbohydrate synthesis and metabolism, and DNA replication, regulation, and repair (Fig 2).

### Genetic distance to other *Ankistrodesmus*

**Fig 3:**
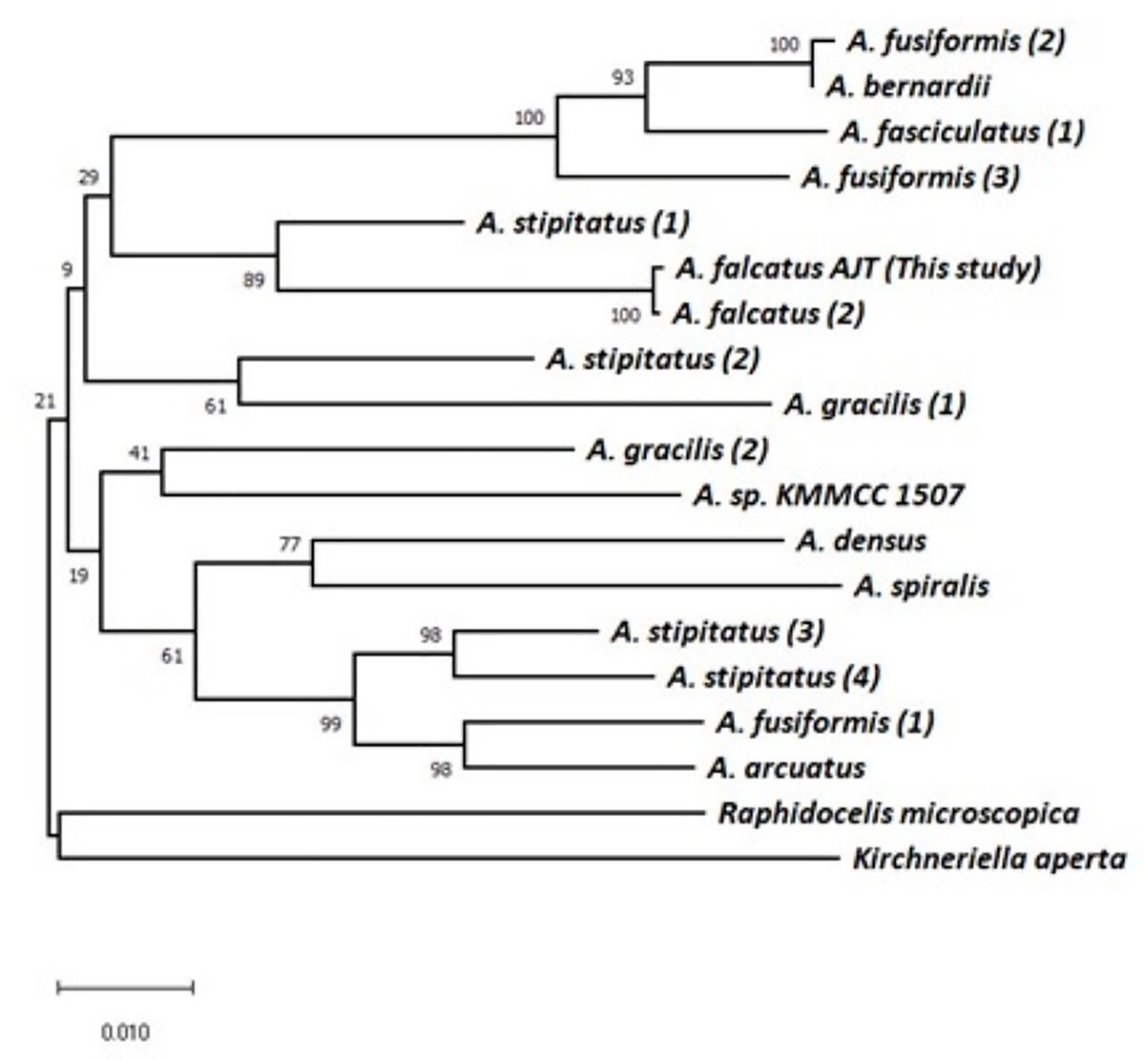
Neighbor-joining tree of genetic distances of rbcL among *Ankistrodesmus* species. Values on nodes indicate bootstrap percentages. Scale bar indicates number of base substitutions per site. Numbers in parentheses indicate different strains of the same species for matching to specific strains in Table 3.

We obtained 16 sequences of rbcL in *Ankistrodesmus* from NCBI (Table 3) and constructed a neighbor-joining tree. We included sequences of two other members of the Selenastraceae, *Raphidocelis microscopica* and *Kirchneriella aperta*. No rbcL sequence was available for *Monoraphidium convolutum* (syn. *A*. *convolutus*) or for *Ankistrodesmus sp*. UCP0001. The rbcL sequence of the AJT strain was virtually identical to that of *A*. *falcatus* UTEX101, and the two sequences grouped together in 100% of bootstrap replicates (Fig 3). *A*. *falcatus* grouped most closely to one strain of *A*. *stipitatus*, but not closely to three other *A*. *stipitatus* strains. In general, deeper nodes were weakly supported, and rbcL distances suggest seven or more similarly related subgroups within *Ankistrodesmus*.

**Table 3:** Taxa, strain, and NCBI accession numbers of sequences used to evaluate genetic distances of rbcL among *Ankistrodesmus*.

Species	Strain	NCBI Accession
*A*. *falcatus*	AJT	this study
*A*. *falcatus (2)*	UTEX 101	JQ394814.1
*A*. *arcuatus*	CCMA 24	KT355740.1
*A*. *bernardii*	CCMA 345	KT833564.1
*A*. *densus*	CCMA 128	KT003371.1
*A*. *fasciculatus*	CB 2012	KT355769.1
*A*. *fusiformis (1)*	CB 2012/6	KT833576.1
*A*. *fusiformis (2)*	CCMA 611	KT833570.1
*A*. *fusiformis (3)*	CCMA 593	KT355761.1
*A*. *gracilis (1)*	CCMA 350	KT003370.1
*A*. *gracilis (2)*	CCMA 005	KT003364.1
*A*. *sp*.	KMMCC 1507	JQ315473.1
*A*. *spiralis*	CB 2012/29	KT833573.1
*A*. *stipitatus (1)*	unknown	KC810299.1
*A*. *stipitatus (2)*	unknown	JX456462.1
*A*. *stipitatus (3)*	SAG 202–5	EF113406.1
*A*. *stipitatus (4)*	CCMA 278	KT355749.1
*Kirchneriella aperta*	SAG 2004	KC145514.1
*Raphidocelis microscopica*	CB 2009/6	KT355768.1

Numbers in parentheses after species names are identifiers to match specific strains to tips in Fig 3.

### Availability of supporting data

Raw sequence data has been deposited in the Sequence Read Archive (SRA) under the accession PRJNA631045. This Transcriptome Shotgun Assembly project has been deposited at DDBJ/EMBL/GenBank under the accession GIOC00000000. The version described in this paper is the first version, GIOC01000000.

## Discussion

Algal transcriptomes are often used for examining the effects of resource availability on growth and physiology at a molecular level, [62–64] for studying microbial interactions and community ecology, [65–68] to explore the molecular mechanisms of algal trophic strategies, [69–72] and to investigate an organism’s potential for biofuel production [73–77].

*A*. *falcatus* is one of the most promising biofuel candidates due to its high lipid productivity compared to other algal species, [9, 10] and is often used as a model for studying how changes to resource availability impacts the lipid content important for biofuel production. For example, Alvarez-Diaz, *et al*. showed that manipulating the concentration of phosphorus or nitrogen and altering the light availability increases *A*. *falcatus’* lipid productivity substantially [78]. George, *et al*. examined how various combinations of media type, light intensity, and photoperiod can influence lipid and biomass yield in *A*. *falcatus* [12], and Kalita, *et al*. (2011) manipulated sodium concentration as a strategy to enhance lipid productivity in the species [79]. The assembled transcriptome presented here will open avenues for deeper investigation into the molecular mechanisms underlying the biochemical and physiological responses in which biofuel industries are interested.

Even though *Ankistrodesmus* species are common freshwater chlorophytes, the phylogenetic relationships of the genus are poorly defined. We used the results from our transcriptome assembly to investigate the genetic distance between *A*. *falcatus* and other *Ankistrodesmus* species with publicly available rbcL sequences. Our neighbor-joining tree is based on a single chloroplast gene and should not be taken as an attempt to identify phylogenetic relationships among *Ankistrodesmus* species. However, it is the best available representation of genetic diversity across the genus and indicates that *A*. *falcatus* is a reasonable representative of *Ankistrodesmus* for genomic purposes. In fact, considering that the generic relationships among the Selenastraceae are poorly resolved and most genera appear to be polyphyletic, [48] *A*. *falcatus* may be a reasonable representative of the whole family.

While discrepancies in assembly statistics are common due to differences in sequencing protocols, assembly methods, and biological variation, [80, 81] our *A*. *falcatus* assembly is comparable to other publicly available, high quality algal transcriptomes with closely-related organisms and similar assembly methods. Chlorophyte transcriptomes range upwards of ~100,000 genes depending on assembly method, with average gene lengths between ~1000-3000bp. Wang, *et al*. assembled the transcriptome of the green algal model *Chlamydomonas reinhardtii* with 91,242 genes, an average contig length of 2,691, and a N50 of 4,554. *Desmodesmus* sp. [82] WR1, another chlorophyte, has been assembled with 32,823 unigenes and a N50 of 1,905bp [83]. A transcriptome assembled for *Scenedesmus acutus* has 51,846 genes with a N50 of 1,351 and an average gene length of 824bp [84]. Yu, *et al*. created an assembly of the chlorophyte *Chlorella minutissima* UTEX2341 which had 14,905 contigs with an average contig length of 2998bp [85]. While their study did not focus on a chlorophyte, Lauritano, *et al*. used a similar assembly pipeline (a combination of Trinity and Velvet/Oases) to create a *de novo* transcriptome for a dinoflagellate which had an average contig length of 1,490bp and N50 of 2,055bp [86]. Our *A*. *falcatus* transcriptome has a total of 17,997 contigs with a N50 of 2,462bp and an average contig length of 1,737bp, which falls within published ranges of expected values for similar unicellular eukaryotes. While we do not have independent information regarding the actual gene lengths of *A*. *falcatus*, our N50 statistic and the average contig length reported here is an improvement over the short N50 (1038bp) and average contig length (508bp) observed in the currently available *Ankistrodesmus sp*. transcriptome. It is possible that the currently available assembly is fragmented or missing information due to differences in sequencing depth and assembly methods, resulting in the shorter average contig lengths and a smaller N50 statistic.

Our BUSCO results suggest that 7.9% of the supposedly single-copy orthologs are duplicated in the *A*. *falcatus* genome. The percent of duplicated BUSCOs is expected to be low because they evolve under single-copy control, but duplication percentages have been shown to range from 1.5% to 13% in other eukaryotes (including *Drosophila melanogaster*, *Caenorhabditis elegans*, *Homo sapiens*, *Lottia gigantea*, *and Aspergillus nidulans;* [47]). Our BUSCO results are comparable to these expectations, and substantially lower than many other available algal transcriptomes, where duplication is reported as high as 52% [87–89]. Because our assembly does not suggest a high level of gene duplication, it indicates that though gene duplications occur, there has not been a whole genome duplication in *Ankistrodesmus*. Duplicated genes offer material for evolutionary forces to act upon, and some duplication events have been linked to stressful environmental conditions in algal species [90, 91]. Selection on these duplicated genes may lead to adaptation within changing environments, and it is possible that the observed, retained gene duplications within the *A*. *falcatus* assembly may be a result of such scenarios.

## Conclusion

Our *A*. *falcatus* transcriptome presented here is of high quality and is an improvement over the currently available *Ankistrodesmus* assembly. Using data that emerged from our sequencing efforts, we created a simple neighbor-joining tree of *Ankistrodesmus* species. This revealed that *A*. *falcatus* appears to be a suitable representative of the Selenastraceae, as well as a good candidate for genomic studies. Though based on limited data, our tree also reinforces prior sequenced-based phylogenies of *Ankistrodesmus* in suggesting the genus is in serious need of taxonomic revision. In both our analysis and other recent reports, distinct strains that are nominally the same species often do not group together. The transcriptome we report here is an important development for studies where community field sample identification may require genomic resources, such as in metagenomic and metatranscriptomic research in freshwater systems where *Ankistrodesmus* species may be prevalent. Additionally, *A*. *falcatus* could potentially be used for biofuel production, and is commonly used as a food source in zooplankton research. This assembly will be valuable to both of these fields as they move further into using genomics and bioinformatics techniques for addressing their central questions.

## References

[1] Brown AH, Weis D (1959) Relation between respiration and photosynthesis in the green alga, *Ankistrodesmus braunii*. Plant Physiol 34(3):224–234. 10.1104/pp.34.3.224 16655207 PMC541181

[2] Shatilov VR, Sof’in AV, Zabrodina TM, Kretovich WL (1982) The role of chloroplast and cytoplasm in the NADP-glutamate dehydrogenase and glutamine synthetase synthesis in *Ankistrodesmus* cells. Molecular and Cellular Biochemistry 49:157–159. 10.1007/BF00231177 6131376

[3] Asselborn V, Fernandez C, Zalocar Y, and Parodi ER (2015) Effects of chlorpyrifos on the growth and ultrastructure of green algae, *Ankistrodesmus gracilis*. Ecotoxical Environ Saf 120:334–341. 10.1016/j.ecoenv.2015.06.015 26099464

[4] Skorupskaite V, Makareviciene V, Ubartas M, Karosiene J, Gumbyte M (2017) Green algae *Ankistrodesmus fusiformis* cell distruption using different modes. Biomass and Bioenergy 107:311–316. 10.1016/j.biombioe.2017.10.015

[5] Guiry MD, Guiry GM. (2021) AlgaeBase. World-wide electronic publication, National University of Ireland, Galway. https://www.algaebase.org.

[6] Marshall HG. (2009) Phytoplankton of the York River. Journal of Coastal Research. 2009: 59–65.

[7] Soylu EN, Gonulol A (2010) Seasonal succession and diversity of phytoplankton in a eutrophic lagoon (Liman Lake). Journal of Environmental Biology 31(5):629–36. 21387913

[8] Maraslioglu F, Soylu EN, Aksoy A (2016) Seasonal succession of the phytoplankton community and evaluation of water quality using trophic diatom index in a stream. Oxidation Communications 39: 459–465.

[9] Griffiths MJ, Harrison STL (2009) Lipid productivity as a key characteristic for choosing algal species for biodiesel production. J. of Applied Phycology 21:439–507. 10.1007/s10811-008-9392-7

[10] Nascimento MD, Dublan MDLA, Ortiz-Marquez JCF, Curatti L (2013) High lipid productivity of an *Ankistrodesmis-Rhizobium* artificial consortium. Bioresour Technol 146: 400–407. 10.1016/j.biortech.2013.07.085 23948276

[11] Nascimento IA, Marques SSI, Cabanelas ITD, Pereira SA, Druzian JI, Souza COD, et al. (2013) Screening microalgae strains for biodiesel production: Lipid productivity and estimation of fuel quality based on fatty acids profiles as selective criteria. BioEnergy Research 6:1–13. 10.1007/s12155-012-9222-2

[12] George B, Pancha I, Desai C, Chokshi K, Paliwal C, Ghosh T, et al. (2014) Effects of different media composition, light intensity and photoperiod on morphology and physiology of freshwater microalgae *Ankistrodesmus falcatus–*A potential strain for bio-fuel production. Bioresource Technology 171: 367–374. 10.1016/j.biortech.2014.08.086 25218209

[13] Singh P, Guldhe A, Kumari S, Rawat I, Bux F (2015) Investigation of combined effect of nitrogen, phosophorus and iron on lipid productivity of microalgae *Ankistrodesmus falcatus* KJ671624 using response surface technology. Biochemical Engineering Journal 94:22–29.

[14] Magdaleno A, Gomez CE, Velez CG, Accorinti J (1997) Preliminary toxicity tests using the green alga, *Ankistrodesmus falcatus*. Environmental Toxicity and Water Quality 12(1):11–14. 10.1002/(SICI)1098-2256(1997)12:1<11::AID-TOX2>3.0.CO;2-B

[15] Martinez-Ruiz E, Martinez-Jeronimo F (2015) Nickel has biochemical, physiological, and structural effects on the green microalga *Ankistrodesmus falcatus*: An integrative study. Aquat Toxicol 169:27–36. 10.1016/j.aquatox.2015.10.007 26513220

[16] Dodson Stanley, (1988), The ecological role of chemical stimuli for the zooplankton: Predator‐avoidance behavior in *Daphnia*. Limnology and Oceanography, 33, 10.4319/lo.1988.33.6part2.1431

[17] Lampert W (2006) *Daphnia*: model herbivore, predator and prey. Polish Journal of Ecology, 54:4:607–620.

[18] Chrislock MF, Sarnelle O, Jernigan LM, Wilson AE (2013) Do high concentrations of microcystin prevent *Daphnia* control of phytoplankton? Water Research, 47(6):1961–1970. 10.1016/j.watres.2012.12.038 23395484

[19] Urrutia-Cordero P, Ekvall MK, Hansson L-A (2016) Controlling Harmful Cyanobacteria: Taxa-Specific Responses of Cyanobacteria to Grazing by Large-Bodied *Daphnia* in a Biomanipulation Scenario. PLoS ONE 11(4): e0153032. 10.1371/journal.pone.0153032 27043823 PMC4820120

[20] Poynton HC, Varshavsky JR, Chang B, Cavigiolio G, Chan S, Holman PS, et al. (2007) Environ. Sci. Technol. 41:1044–1050. 10.1021/es0615573 17328222

[21] Neves M, Castro BB, Vidal T, Vieira R, Marques JC, Coutinho JAP, et al. (2015) Biochemical and populational responses of an aquatic bioindicator species, *Daphnia longispina*, to a commercial formulation of a herbicide (Primextra Gold TZ) and its active ingredient (S-metolachlor). Ecological Indicators, 53:220–230.

[22] Goulden CE, Hornig LL (1980) Population oscillations and energy reserves in planktonic Cladocera and their consequences to competition. Population Biology 77(3):1716–1720. https://dx.doi.org/10.1073%2Fpnas.77.3.1716 10.1073/pnas.77.3.1716 PMC348568 16592788

[23] Tessier AJ NL Consolatti (1991) Resource quantity and offspring quality in *Daphnia*. Ecology 72:468–478. 10.2307/2937188

[24] DeMott WR (2003) Implications of element deficits for zooplankton growth. *Hydrobiologia* 491:177–184.

[25] Steiner CF, Klausmeier CA, Litchman E. (2012) Transient dynamics and the destabilizing effects of prey heterogeneity. Ecology 93(3): 632–644. 10.1890/10-2320.1 22624217

[26] DeMott WR, Van Donk E (2013) Strong interactions between stoichiometric constraints and algal defenses: evidence from population dynamics of *Daphnia* and algae in phosphorus-limited microcosms. Oecologia 171: 175–186. 10.1007/s00442-012-2404-y 22802021 PMC3538120

[27] Dudycha JL (2003) A multi-environment comparison of senescence between sister species of *Daphnia*. Oecologia 136: 141–147. 10.1007/s00442-003-1246-z 16228255

[28] Dudycha JL, Hassel C (2013) Aging in sexual and obligately asexual clones of *Daphnia* from temporary ponds. Journal of Plankton Research 35: 253–259. 10.1093/plankt/fbt008 23467752 PMC3589896

[29] Kim E, Ansell C, Dudycha JL (2014) Resveratrol and food effects on lifespan and reproduction in the model Crustacean *Daphnia*. J Exp Zool 321A:48–56. 10.1002/jez.1836 24133070 PMC4103430

[30] Hall SR, Knight CJ, Becker CR, Duffy MA, Tessier AJ, Cáceres CE (2009) Quality matters: resource quality for hosts and the timing of epidemics. Ecology Letters 12:118–128. 10.1111/j.1461-0248.2008.01264.x 19049510

[31] Searle CL, Mendelson JR, Green LE, Duffy MA (2013) *Daphnia* predation on the amphibian chytrid fungus and its impacts on disease risk in tadpoles. Ecology & Evolution 3:4129–4138. 10.1002/ece3.777 24324864 PMC3853558

[32] Penczykowski RM, Lemanski BCP, Sieg RD, Hall SR, Ochs JH, Kubanek J, et al. (2014) Poor resource quality lowers transmission potential by changing foraging behavior. Funct Ecol 28:1245–1255. 10.1111/1365-2435.12238

[33] Auld SKJR, Searle CL, and Duffy MA (2017) Parasite transmission in a natural multihost-multiparasite community. Philos Trans R Soc B Biol Sci 372:20160097. 10.1098/rstb.2016.0097 PMC5352823 28289264

[34] Brandon C, Dudycha JL (2014) Ecological constraints on sensory systems: compound eye size in *Daphnia* is reduced by resource limitation. J Comp Physiol A 200:749–758. 10.1007/s00359-014-0918-y 24865992

[35] Sarnelle O, Wilson AE (2005) Local adaptation of *Daphnia pulicaria* to toxic cyanobacteria. Limnology and Oceanography 50:1565–1570. 10.4319/lo.2005.50.5.1565

[36] Allen MR., Thum RA, Vandyke JN, Cáceres CE (2012) Trait sorting in *Daphnia* colonising man-made lakes. Freshw. Biol. 57:1813–1822. 10.1111/j.1365-2427.2012.02840.x

[37] Rogalski MA (2017) Maladaptation to acute metal exposure in resurrected *Daphnia ambigua* clones after decades of increasing contamination. American Naturalist 189: 443–452. 10.1086/691077 28350505

[38] Rogalski MA, Duffy MA (2020) Local adaptation of a parasite to solar radiation impacts disease transmission potential, spore yield, and host fecundity. Evolution 74: 1856–1864. 10.1111/evo.13940 32052425

[39] Martinez-Jeronimo F (2012) Description of the individual growth of *Daphnia magna* (Crustacea: Cladocera) through the von Bertalanffy growth equation. Effect of photoperiod and temperature. Limnology 13:65–71. 10.1007/s10201-011-0356-2.

[40] Kilham S, Kreeger D, Goulden C, Lynn S (1997) Effects of algal food quality on fecundity and population growth rates of *Daphnia*. Freshwater Biology 38:638–647.

[41] Dudycha JL, Brandon C, Deitz K (2011) Population genomics of resource exploitation: insights from gene expression profiles of two *Daphnia* ecotypes fed alternate resources. Ecology and Evolution 2(1). 10.1002/ece3.30 PMC3298946 22423327

[42] Schumpert CA, Dudycha JL, Patel RC (2015) Development of an efficient RNA interference method by feeding for the microcrustacean *Daphnia*. BMC Biotechnology 15(91). 10.1186/s12896-015-0209-x 26446824 PMC4597761

[43] Schumpert CA, Nelson J, Kim E, Anderson C, Dudycha JL, Patel RC (20152016) Involvement of *Daphnia pulicaria* Sir2 in regulating stress response and lifespan. Aging 8: 402–417. 10.18632/aging.100909 26978617 PMC4789591

[44] Castro JC, Maddox JD, Paredes JD, Rodriguez HN, Aguilar CP, Marapara JL, et al. (2017) De novo RNA-Seq analysis of the oleaginous microalgae *Ankistrodesmus sp*. UCP0001: Gene identification and metabolic pathways reconstruction for the biosynthesis of fatty acids and triaglyglycerols. Plant Cell Biotechnology and Molecular Biology 18(5–6):219–230.

[45] Thanh T, Chi V, Abdullah MP, Omar H, Noroozi M, Ky H, Napis S (2011) Construction of cDNA library and preliminary analysis of expressed sequence tags from green microalga *Ankistrodesmus convolutus* Corda. Mol Biol Rep 38:177–182. 10.1007/s11033-010-0092-4 20354903

[46] Thanh T, Chi V, Abdullah MP, Omar H, Noroozi M, Napis S (2011) Cloning and characterization of ribulose-1,5-bisphosphate carboxylase/oxygenase small subunit (*RbcS)* cDNA from green microalga *Ankistrodesmus convolutus*. Mol Biol Rep 38:5297–5305. 10.1007/s11033-011-0679-4 21287365

[47] Krienitz L., Ustinova I., Friedl T. and Huss V.A.R. (2001) Traditional generic concepts versus 18S rRNA gene phylogeny in the green algal family Selenastraceae (Chlorophyceae: Chlorophyta). Journal of Phycology, 37: 852–865.

[48] Garcia da Silva TG, Bock C, Sant’Anna CL, Bagatini IL, Wodniok S, Vieira AAH (2017) Selenastraceae (Sphaeropleales, Chlorophyceae): rbcL, 18s rDNA and ITS-2 secondary structure enlightens traditional taxonomy, with description of two new genera, *Messastrum gen*. *nov*. and *Curvastrum gen*. *nov*. Fottea. 17(1):1–19.

[49] Komárková-Legnerová J. (1969). The systematics and ontogenesis of the genera *Ankistrodesmus* Corda and *Monoraphidium* gen. nov. In: *Studies in Phycology*. (Fott B. Ed), pp. 75–144. Prague: Academia Publishing House of the Czechosolvak Academy of Sciences.

[50] Gorham RP R.P., McLachlan J J., Hammer UT U.T., Kim WK W.K. (1964) Isolation and culture of toxic strains of Anabaena flos-aquae (Lyngb.) de Bréb Verh. Int. Ver. Theor. Angew. Limnol., 15: (1964), pp. 796–804.

[51] Andrews S (2010) FastQC: a quality control tool for high throughput sequence data. Babraham Institute. http://www.bioinformatics.babraham.ac.uk/projects/fastqc/. Accessed 25 Jan 2020.

[52] Bolger AM, Lohse M, Usadel B (2014) Trimmomatic: A flexible trimmer for Illumina Sequence Data. Bioinformatics 30(15):2114–20. 10.1093/bioinformatics/btu170 24695404 PMC4103590

[53] Grabherr MG, Haas BJ, Yassour M, Levin JZ, Thompson DA, Amit I, et al. (2011) Full-length transcriptome assembly from RNA-seq data without a reference genome. Nat Biotechnol, 29(7):644–652. 10.1038/nbt.1883 21572440 PMC3571712

[54] Schulz MH, Zerbino DR, Vingron M, Birney E (2012) Oases: Robust de novo RNA-seq assembly across the dynamic range of expression levels. Bioinformatics 28(8):1086–92. 10.1093/bioinformatics/bts094 22368243 PMC3324515

[55] Gilbert D (2013) EvidentialGene:tr2aacds, mRNA transcript assembly software. http://arthropods.eugenes.org/EvidentialGene/about/EvidentialGene_trassembly_pipe.html Accessed 26 May 2020.

[56] Quast C, Pruesse E, Yilmaz P, Gerken J, Schweer T, Yarza P, et al. (2013) The SILVA ribosomal RNA gene database project: Improved data processing and web-based tools. Nucl Acids Res 41(D1): D591–596. 10.1093/nar/gks1219 23193283 PMC3531112

[57] Simao FA, Waterhouse RM, Ioannidis P, Kriventseva EV, Zdobnov EM (2015) BUSCO: Assessing genome assembly and annotation completeness with single-copy orthologs. Bioinformatics 31(19):3210–2. 10.1093/bioinformatics/btv351 26059717

[58] Smith-Unna RD, Boursnell C, Patro R, Hibberd JM, Kelly S (2016) TransRate: Reference free quality assessment of de-novo transcriptome assemblies. Genome Research 26(8):1134–114. 10.1101/gr.196469.115 27252236 PMC4971766

[59] Scott C (2018) Dammit: an open and accessible de novo transcriptome annotator. https://dammit.readthedocs.io/en/refactor-1.0/. Accessed 26 May 2020.

[60] Kumar S., Stecher G., Li M., Knyaz C., and Tamura K. (2018). MEGA X: Molecular Evolutionary Genetics Analysis across computing platforms. *Molecular Biology and Evolution* 35:1547–1549. 10.1093/molbev/msy096 29722887 PMC5967553

[61] Saitou N. and Nei M. (1987). The neighbor-joining method: A new method for reconstructing phylogenetic trees. *Molecular Biology and Evolution* 4:406–425. 10.1093/oxfordjournals.molbev.a040454 3447015

[62] Harke MJ, Juhl AR, Haley ST, Alexander H, Dyhrman ST (2017) Conserved transcriptional responses to nutrient stress in bloom-forming algae. Front Microbiol 8:1279. 10.3389/fmicb.2017.01279 28769884 PMC5513979

[63] Im S, Lee HN, Jung HS, Yang S, Park EJ, Hwang MS, et al. (2017) Transcriptome-based identification of the desiccation response genes in marine red algae *Pyropia tenera* (Rhodophyta) and enhancement of abiotic stress tolerance by *PtDRG2* in *Chlamydomonas*. Mar Biotechnol (NY) 19:232–245. 10.1007/s10126-017-9744-x 28421378

[64] Zhang Z, Sun D, Chen F (2020) Comparative transcriptome analysis revealing the mechanisms underlying light-induced total fatty acid and carotenoid accumulation in *Crypthecodinium* sp. SUN. Algal Research 47. 10.1016/j.algal.2020.101860

[65] Schwartz JA, Curtis NE, Pierce SK (2010) Using algal transcriptome sequences to identify transferred genes in the sea slug, *Elysia chlorotica*. Evolutionary Biology 37:29–37. 10.1007/s11692-010-9079-2

[66] Meron D, Maor-Landaw K, Weizman E, Ben-Asher HW, Eyal G, Banin E, et al. (2019) The algal symbiont modifies the transcriptome of the Scleractinian coral *Euphyllia paradivisa* during heat stress. Microorganisms 7(8):256. 10.3390/microorganisms7080256 31409030 PMC6723837

[67] Metegnier G, Paulino S, Ramond P, Siano R, Sourisseau M, Destombe C, Gac ML (2020) Species specific gene expression dynamics during harmful algal blooms. Sci Rep 10:6182. 10.1038/s41598-020-63326-8 32277155 PMC7148311

[68] Vorobev A, Dupouy M, Carradec Q, Delmont TO, Annamale A, Wincker P, et al. (2020) Transcriptome reconstruction and functional analysis of eukaryotic marine plankton communities via high-throughput metagenomics and metatranscriptomics. Genome Res. 30: 647–659. 10.1101/gr.253070.119 32205368 PMC7197479

[69] Koid AE, Liu Z, Terrado R, Jones AC, Caron DA, Heidelberg KB (2014) Comparative transcriptome analysis of four Prymnesiophyte algae. *PLoS ONE* 9(6):e97801. 10.1371/journal.pone.0097801 24926657 PMC4057078

[70] Liu Z, Jones AC, Campbell V, Hambright KD, Heidelberg KB, Caron DA (2015) Gene expression in the mixotrophic prymnesiophyte, *Prymnesium parvum*, responds to prey availability. Front Microbiol 6:319. 10.3389/fmicb.2015.00319 25941521 PMC4403553

[71] Caron DA, Alexander H., Allen AE, Archibald JM, Armbrust EV, Bachy C, et al. (2016) Probing the evolution, ecology and physiology of marine protists using transcriptomics. Nat Rev Microbiol 15: 6–20. 10.1038/nrmicro.2016.160 27867198

[72] Beisser D, Graupner N, Bock C, Wodniok S, Grossmann L, Vos M, et al. (2017) Comprehensive transcriptome analysis provides new insights into nutritional strategies and phyologenetic relationships of chrysophytes. PeerJ Life and Environment 10.7717/peerj.2832 PMC5228505 28097055

[73] Davidson JO, Overton J, Waikel R (2012) Transcriptome analysis of *Chlorella protothecoides* to identify novel pro-lipid genes for biofuel production. The FASEB Journal 26.

[74] Lv H, Qu G, Qi X, Lu L, Tian C, Ma Y (2013) Transcriptome analysis of *Chlamydomonas reinhardtii* during the process of lipid accumulation. Genomics 101(4):229–237. 10.1016/j.ygeno.2013.01.004 23396177

[75] Chang WC, Zheng HQ, Chen CNN (2016) Comparative transcriptome analysis reveals a potential photosynthate partitioning mechanism between lipid and starch biosynthetic pathways in green microalgae. Algal Research 16:54–62. 10.1016/j.algal.2016.03.007

[76] Huang W, Ye J, Zhang J, Lin Y, He M, Huang J (2016) Transcriptome analysis of *Chlorella zofingiensis* to identify genes and their expressions involved in astaxanthin and triacylglycerol biosynthesis. Algal Research 17:236–243. 10.1016/j.algal.2016.05.015

[77] Sharma T, Chauhan RS (2016) Comparative transcriptomics reveals molecular components associated with differential lipid accumulation between microalgal sp., *Scenedesmus dimorphus* and *Scenedesmus quadricauda*. Algal Research 19: 109–122. 10.1016/j.algal.2016.07.020

[78] Alvarez-Diaz PD, Ruiz J, Arbib Z, Barragan J, Garrido-Perez C, and Perales JA (2014) Lipid production of microalga *Ankistrodesmus falcatus* increased by nutrient and light starvation in a two-stage cultivation process. Applied Biochem And Biotech 174:1471–1783. 10.1007/s12010-020-03335-5 25119548

[79] Kalita N, Baruah G, Goswami RCD, Talukdar J, Kalita MC (2011). *Ankistrodesmus falcatus*: A promising candidate for lipid production, its biochemical analysis and strategies to enhance lipid productivity. J. of Micro. And Biotech. Research. 1(4):148–157.

[80] Hornett EA, Wheat CW (2012) Quantitative RNA-Seq analysis in non-model species: assessing transcriptome assemblies as a scaffold and the utility of evolutionary divergent genomic reference species. BMC Genomics 13:361. 10.1186/1471-2164-13-361 22853326 PMC3469347

[81] O’Neil ST, Emrich SJ (2013) Assessing de novo transcriptome assembly metrics for consistency and utility. BMC Genomics 14:465. 10.1186/1471-2164-14-465 23837739 PMC3733778

[82] Wang Ning; Qian Zhixin; Luo Manwei; Fan Shoujin; Zhang Xuejie; Zhang Luoyan. 2018. "Identification of Salt Stress Responding Genes Using Transcriptome Analysis in Green Alga Chlamydomonas reinhardtii" Int. J. Mol. Sci. 19, no. 11: 3359. 10.3390/ijms19113359 PMC6274750 30373210

[83] Sirikhachornkit A., Suttangkakul A., Vuttipongchaikij S. et al. De novo transcriptome analysis and gene expression profiling of an oleaginous microalga *Scenedesmus acutus* TISTR8540 during nitrogen deprivation-induced lipid accumulation. Sci Rep 8, 3668 (2018). 10.1038/s41598-018-22080-8 29487383 PMC5829077

[84] Wang R, Diao P, Chen Q, Wu H, Xu N, Duan S. 2017. Identification of novel pathways for biodegradation of bisphenol A by the green alga *Desmodesmus* sp. WR1, combined with mechanistic analysis at the transcriptome level. *Chemical Engineering Journal*. 321. 424–431.

[85] Yu M, Yang S, Lin X (2015) De-novo assembly and characterization of *Chlorella minutissima* UTEX2341 transcriptome by paired-end sequencing and the identification of genes related to the biosynthesis of lipids for biodiesel. Marine Genomics 25: 69–74. 10.1016/j.margen.2015.11.005 26590019

[86] Lauritano C, Luca DD, Ferrarini A, Avanzato C, Minio A, Esposito F, et al. (2017) De novo transcriptome of the cosmopolitan dinoflagellate *Amphidinium carterae* to identify enzymes with biotechnological potential. Sci Rep 7:11701. 10.1038/s41598-017-12092-1 28916825 PMC5601461

[87] Ogura A, Akizuku Y, Imoda H, Mineta K, Gojobori T, Nagai S (2018) Comparative genome and transcriptome analysis of diatom, *Skeletonema costatum*, reveals evolution of genes for harmful algal bloom. BMC Genomics 19:765. 10.1186/s12864-018-5144-5 30348078 PMC6198448

[88] Nam O, Park JM, Lee H, Jin E (2019) De novo transcriptome profile of coccolithophorid alga *Emiliania huxleyi* CCMP371 at different calcium concentrations with proteome analysis. PLoS ONE 14(8): e0221938. 10.1371/journal.pone.0221938 31465514 PMC6715215

[89] Alsenani F, Wass TJ, Ma R, Eltanahy E, Netzel ME, and Schenk PM (2019) Transcriptome-wide analysis of *Chlorella* reveals auxin-induced carotenogensis pathway in green microalgae. Algal Research 37: 320–335. 10.1016/j.algal.2018.12.002

[90] Wu G, Hufnagel DE, Denton AK, Shiu SH (2015) Retained duplicate genes in green alga *Chlamydomonas reinhardtii* tend to be stress responsive and experience frequent response gains. BMC Genomics 16(1):149. 10.1186/s12864-015-1335-5 25880851 PMC4364661

[91] Qiao X, Li Q, Yin H, Qi K, Li L, Wang R, et al. (2019) Gene duplication and evolution in recurring polyploidization-diploidization cycles in plants. Genome Biology 20:38. 10.1186/s13059-019-1650-2 30791939 PMC6383267

